# Mobile App Intervention Increases Adherence to Home Exercise Program After Whiplash Injury—A Randomized Controlled Trial (RCT)

**DOI:** 10.3390/diagnostics14232729

**Published:** 2024-12-04

**Authors:** Blaž Barun, Zdravko Divić, Dušanka Martinović Kaliterna, Ana Poljičanin, Benjamin Benzon, Jure Aljinović

**Affiliations:** 1 Division of Physical Medicine and Rehabilitation with Rheumatology, University Hospital of Split, 21000 Split, Croatia; blaz.barun@mefst.hr (B.B.); jure.aljinovic@mefst.hr (J.A.); 2 University of Split School of Medicine, 21000 Split, Croatia; 3 Department of Health Studies, University of Split, 21000 Split, Croatia; 4 Departments of Anatomy, Histology and Embryology, School of Medicine, University of Split, 21000 Split, Croatia

**Keywords:** randomized controlled trial, whiplash injury, adherence to exercise, physical therapy, Neck Disability Index

## Abstract

Objective: Can mobile app intervention via push notifications increase adherence to exercise and reduce disability and pain after a whiplash injury? Methods: A randomized controlled trial was conducted with concealed allocation, blinding of some assessors, and an intention-to-treat analysis. Participants who sustained whiplash injury at most 3 months prior were divided into active and control groups. Both groups completed a two-part physiotherapist-supervised physical therapy program (3-week break in between, ten sessions each, 5x/week). The program included TENS, therapeutic ultrasound, and exercises (breathing, ROM, deep neck flexor activation, and stretching). Both groups were encouraged to exercise at home. The active group additionally received push notifications through the mobile app once a day as a reminder to exercise. Outcomes were adherence to exercise (four-point Likert scale), physical functioning (NDI), pain intensity (VAS), perceived recovery (three-point Likert scale), work information, psychological functioning (PCS), and HRQoL (SF-12) at baseline and 6-month follow-up. Results: At month 6, when comparing the groups, the intervention group showed higher adherence to home exercise (3 [2–4] vs. 2 [2–4]; p = 0.005, median [IQR]) and improved HRQoL (∆SF-12) (20 [6–36] vs. 15 [9–23]; p = 0.038). Unlike the control group, the intervention group showed a significant decrease in pain catastrophizing (31%; p = 0.01). A multivariant analysis showed that mobile app intervention influenced adherence most (≈1 Likert point). The groups did not differ in NDI, pain VAS, perceived recovery, or work limitation. Conclusions: Mobile app intervention increased adherence to home exercise, reduced pain catastrophizing, and increased HRQoL six months after a whiplash injury. Trial registration: ClinicalTrials.gov NCT05704023.

## 1. Introduction

Neck pain is one of the leading causes of disability [1]. Whiplash injury is the most common injury related to motor vehicle accidents, and up to 50% of injuries lead to chronic neck pain and disability [2,3]. Besides being a cause of chronic physical impairment, it harms mental health, leading to higher healthcare system use and lower productivity, resulting in an increased burden on society [4]. The initial level of disability measured by the Neck Disability Index (NDI) [5] is recognized as a critical risk factor for developing chronic disability [6,7,8]. High initial pain intensity, coping styles, depression, fear of movement, and catastrophizing are other contributing factors for higher disability levels [9,10,11,12,13,14]. Conservative treatments following a whiplash injury usually include a variety of active (exercises) and passive (rest, ultrasound, transcutaneous electric nerve stimulation (TENS), and laser therapy) procedures. Although conservative treatments are the golden standard after a whiplash injury, their effectiveness is still not statistically proven [15]. However, a positive effect of education and exercise was reported to improve pain and disability levels after a whiplash injury [16,17].

With the widespread use of smartphones and the development of mobile apps, many studies are now investigating ways to utilize these technological advances in medicine. Mobile apps can be useful in monitoring health status and providing feedback and health information in real time. Papers describing the promotion of health by stimulating an increase in physical activity, improvements in dietary habits, a reduction in smoking, and the monitoring of diabetes can be found in the literature [18,19,20,21]. Likewise, research has been conducted in the physical and rehabilitation medicine field where mobile apps have been shown to have positive effects on functional and health outcomes (gait, mobility, pain, and quality of life) [22]. Furthermore, mobile apps are already proven to be effective in increasing adherence in cardiac rehabilitation and could also help in other conditions where adherence to intervention is an issue, such as neck pain, including whiplash injury of the neck [23,24].

No clinical trials or systematic reviews regarding increasing adherence to exercise through mobile apps after whiplash injury of the neck are found in medical databases. The effect of adherence to exercise and disability level, comparing standard written recommendations to mobile apps with push notifications, is not yet known.

The aim of this randomized controlled trial was to determine whether the mobile app “WIApp” can be effective in promoting adherence to exercise and consequently lead to better recovery of patients after whiplash injury of the neck.

## 2. Methods

### 2.1. Study Design

This is a randomized, active-controlled, parallel-group unmasked to intervention allocation trial, which primarily assessed the effects of adding a mobile app to standard treatments on the adherence and recovery of patients following a whiplash injury. Standard care supported by a mobile reminder app (Arm A) was compared to standard care alone (Arm B). A timeline of data collection is provided in Additional File 1: Supplementary Figure S1.

### 2.2. Participants

#### 2.2.1. Eligibility Criteria

The enrolled participants met the following inclusion criteria: age ≥ 18 years, whiplash injury of the neck in a car accident as the driver or co-driver, whiplash injury of the neck diagnosed by a physical and rehabilitation medicine specialist within three months, an NDI score higher than 5 (10%), possession of and ability to use a smartphone and a mobile app, and signed written informed consent. Subjects were excluded if they had an accident in any other type of vehicle other than a car, sustained a bone fracture or spinal cord injury in the accident, treated a malignant disease in the last five years, or non-compliance was expected (it was not possible to use a smartphone because of severe mental or physical impairment).

#### 2.2.2. Settings and Locations

This study was conducted at the Division of Physical Medicine and Rehabilitation with Rheumatology, University Hospital of Split, Croatia.

### 2.3. Intervention Description

This study comprised two groups: a “WIApp” intervention group and a control group. A physical therapy specialist examined all participants, referred them to the physical therapy program, and allocated them to either the intervention or control group. If needed, pain medications were prescribed. In between and after the physical therapy program, patients exercised at home with or without mobile app support.

#### 2.3.1. Physical Therapy

All participants undertook the standardized physical therapy program guided by a physiotherapist. The detailed physical therapy program was described in our previous paper [8].

A two-part physical therapy program was carried out: a 2-week program with therapy 5 times a week, followed by a 3-week break, and then another 2 weeks of therapy.

#### 2.3.2. Mobile App (Intervention Group)

WIApp is a smartphone app that aims to improve patient adherence to exercise and help patients recover after a whiplash injury; it includes a daily reminder to exercise, photographs, and explanations of exercises. The app was developed by the professional Mateh software d.o.o. from Zagreb, Croatia, and was available for iOS and Android. The main feature of WIApp is exercise support. Daily, at 7 p.m., patients received a notification with a reminder to exercise. The app included photographs of the exercises, with instructions, that patients could look at and read if they forgot which exercises they needed to perform and how.

After the participants met the eligibility criteria and were included in the trial, the physician referred them to physical therapy. After completion of the first part of the physical therapy program and allocation to the intervention group, they were introduced to the WIApp by the physician who, also being the administrator of WIApp, created a user profile for the participant. The participant downloaded the WIApp to their mobile phone and accessed their profile using a QR code or 16-digit code. The physician explained to the patient how they need to use the app during the follow-up.

#### 2.3.3. Control Group

After the participants completed the first and second parts of the physical therapy program, a physiotherapist determined whether one could continue performing exercises at home. After participants confirmed that they knew how to perform the exercises, the physician gave them written and illustrated material explaining the home exercise program (HEP). Participants were advised to continue the HEP and to record adherence on a weekly basis.

### 2.4. Outcomes

We used the core outcome set according to Recommendations For Core Outcome Domain Set For Whiplash-Associated Disorders (CATWAD) [25]. Measurements were undertaken at two time points in each group: at baseline and 6-month follow-up. The only discrepancy between the registered protocol and that described in this paper was a change of adherence to exercise from a secondary to primary outcome.

1. Adherence to exercise: assessed at the 6-month follow-up with a four-point Likert scale (EAS) regarding weekly exercise completion (classified as no sessions, occasional, 2–4 sessions/week, or ≥5 sessions/week);
2. Physical functioning: assessed before physical therapy and at the 6-month follow-up with an NDI (where values of 0–8% are regarded as no disability, 10–28% as mild disability, 30–48% as moderate disability, 50–68% as severe disability, and 70–100% as complete disability);
3. Perceived recovery: assessed before physical therapy and at the 6-month follow-up with a three-point Likert-scale (PRS) (where 1 indicates non-recovery and 3 indicates full recovery);
4. Work: assessed before physical therapy and at the 6-month follow-up with work status information, work-time loss, and a work limitation scale (WLS) (a six-point Likert scale where 1 indicates normal work capability and 6 indicates no working capability);
5. Psychological functioning: assessed before physical therapy and at the 6-month follow-up with a Pain Catastrophizing Scale (PCS) (score range from 0 to 50, a score of 30 or more represents a clinically significant level of catastrophizing);
6. Health-related quality of life (HRQoL) and social functioning: assessed before physical therapy and at the 6-month follow-up with a Short form-12 (SF-12) Health Survey version 1 (online scoring calculator: https://orthotoolkit.com/sf-12/) and Social Functioning Scale (SFS)—a five-point Likert scale where 1 indicates a constant limitation in social activities and 5 indicates none limitation;
7. Pain intensity (neck region and head): assessed before physical therapy and at the 6-month follow-up with a visual analog scale (VAS) (ranging from 0  =  no pain to 10  =  maximum pain).

### 2.5. Sample Size

For the sample size calculation, we used the usual rule of thumb, which states that 10 to 30 participants per variable are needed for modeling. Therefore, we planned to analyze 60 participants. Due to potential dropouts, we planned to enroll 68 participants.

### 2.6. Assignment of Interventions: Allocation

#### 2.6.1. Sequence Generation

The patients were randomized with an online randomization tool (randomizer.org) in a 1:1 ratio to receive either standard care supported by a reminder app (Arm A) or standard care (Arm B) for treatment of whiplash injury.

#### 2.6.2. Allocation Concealment

To ensure allocation concealment, one research team member created a randomization list and another allocated the participants to a group.

#### 2.6.3. Implementation

Generation of the allocation sequence, enrolment of participants, and assignment of participants to interventions were performed by physicians registered as investigators for this trial.

### 2.7. Blinding

Due to the nature of interventions applied in this study, blinding of the participants and outcome assessors was not possible. However, the physiotherapists and data analysts were blinded.

### 2.8. Statistical Methods

Continuous data are presented as median ± interquartile range (IQR), or mean and SD, and proportions are presented as percentages. Differences in continuous variables were tested with Mood’s median test, Mann–Whitney tests, and t-tests. Differences in proportions were tested by Fisher’s exact test. Statistical measures of evidence are presented as an effect size and its standard error, *p* values, R^2^, and evidence ratio based on differences in corrected Akaike information criteria (AIC). For the multivariate analysis and correlation, we used a linear model. Statistical analyses were performed in Past3 Software and GraphPad Prism 10.0 software.

## 3. Results

**Figure 1:**
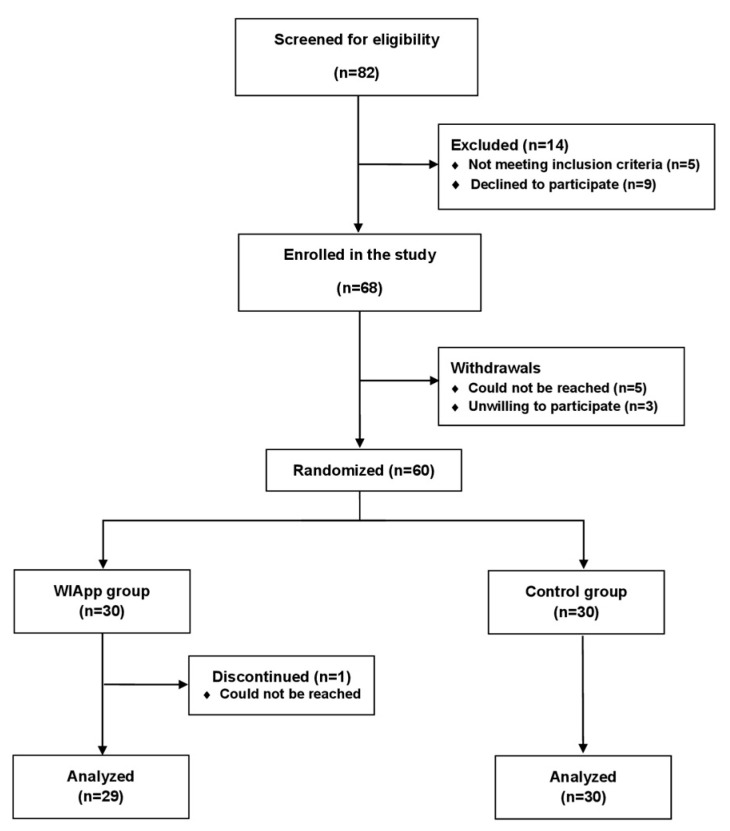
Flow of participants through the trial.

A total of 78 participants were screened for the study; 68 were enrolled, and 60 were randomized into 2 treatment groups. The screening, randomization, and follow-up are summarized in Figure 1. The participants were recruited between January 2023 and February 2024 with a 6-month follow-up period.

The baseline characteristics were similar between the groups (Table 1). The median age (IQR) in the WIApp group was 37 (24–51), and 19 participants (66%) were female.

Fifty-nine participants completed the study and were analyzed: 29 in the WIApp group and 30 in the control group. Table 2 summarizes all measured outcomes at baseline and the 6-month follow-up for both groups.

The WIApp group showed significantly higher adherence to HEP, with most participants reporting exercising 2–4 times/week, compared to the control group, who reported occasional exercise (*p* = 0.005). The level of adherence to exercise for both groups is shown in Table 3.

Adherence correlated differently, i.e., oppositely, with initial NDI% in the intervention group (r = −0.18) and control group (r = 0.26) (*p* < 0.001). Using a multivariant analysis, we calculated how particular variables (initial NDI%, initial pain VAS, initial PCS, and intervention) influenced exercise adherence. The model that considers mobile app intervention describes data 17 times better (∆AIC = 5.645, ER ≈ 16.82, *p* = 0.007, and R^2^ = 23%) than the one without it (Table 4).

Initially, 52% of patients in the WIApp and 43% in the control group reported moderate disability (Table 3), with no difference between the groups (*p* = 0.6). At the six-month follow-up, both groups had significantly lower NDI% scores, with 48% of participants in the intervention and 53% in the control group reporting mild disability. Furthermore, the median change in the NDI% was not statistically different between the groups (20 IQR 10–27 vs. 17 IQR 6–25; *p* = 0.516).

When analyzing PRS six months after the injury, most participants reported partial recovery (16, [55] vs. 17 [57]), followed by total recovery (11, [38] vs. 12, [40]) and no recovery (2, [7] vs. 1 [3]), with no significant difference between the groups (*p* = 0.823).

At baseline, significant pain catastrophizing was reported in 41% of participants in the WIApp and 31% in the control group (*p* = 0.58). Six months after the injury, pain catastrophizing was decreased by 31% in the WIApp group (*p* = 0.01) and by 18% in the control group (*p* = 0.12). Additionally, at baseline, we found that increased pain catastrophizing was connected to increased NDI% (OR = 1.071, 95%CI 1.025 to 1.127; *p* = 0.002), and a similar connection was found six months after the injury (OR = 1.111, 95%CI 1.049 to 1.2; *p* < 0.001).

Initially, 91% of participants in the WIApp and 87% in the control group reported moderate or severe pain intensity levels (Table 3), with no difference between the groups (*p* = 0.369). Both groups reported significantly lower average pain intensity levels six months after the injury, with 45% of participants in the intervention and 50% in the control group reporting mild pain. No difference in the average change of pain VAS was found between groups (1.45 ± 2.4 vs. 1.45 ± 1.95; *p* = 0.9).

After the injury, absenteeism was reported in 79% of participants in the WIApp and 91% in the control group (*p* = 0.389), with only 3% of participants from both groups having no work limitations (Table 3). Six months after the injury, participants had significantly fewer work limitations, with 38% of subjects in the intervention and 30% in the control group reporting no limitation. The median change in work limitation (ΔWLS) showed no difference between the groups (1 IQR 0–1.5 vs. 1 IQR 0–2; *p* = 0.770). Although there was a difference in the median workday loss between the groups, it was not statistically significant (12 IQR 0–58 vs. 24 IQR 10–70; *p* = 0.1).

After the injury, 45% of participants in the WIApp and 47% in the control group reported they sometimes have limitations in social activities (Table 3), with no difference between the groups (*p* = 0.758). Six months after the injury, both groups had limitations in social activities significantly less of the time, with 41% of participants in the intervention and 43% in the control group reporting only occasional limitations in social activities. There was no difference in the change in social limitation (ΔSFS) between the groups (1 IQR 0–1 vs. 1 IQR 0–1 *p* = 0.970).

There was no difference in the initial median SF-12 scores (40 IQR 36–50 vs. 49 IQR 36–57; *p* = 0.12). Both groups reported significantly higher SF-12 scores six months after the injury (60 IQR 48–83; *p* < 0.001 vs. 66 IQR 45–78; *p* = 0.01). When the change in the SF-12 was compared between groups, the intervention group showed a significantly greater increase (20 IQR 6–36 vs. 15 IQR 9–23; *p* = 0.038).

## 4. Discussion

This study compared the effectiveness of adding mobile app intervention to standard physical therapy in increasing adherence to exercise and lowering disability in a 6-month follow-up period in patients after whiplash injury of the neck. Mobile app use increased adherence to HEP. Both groups showed significant improvement in disability, pain, social functioning, and work capabilities, with no significant differences between them. Pain catastrophizing was significantly decreased, and HRQoL improved more in the intervention group six months after the injury.

HEP for neck pain showed effectiveness in decreasing pain and disability [26]. Furthermore, adherence to HEP plays a key role in optimizing the benefits of exercise, and it is connected to better functional outcomes [27]. Adherence to HEP is challenging for patients, especially when it is needed for a prolonged period, as it is after a whiplash injury [28,29]. Digital interventions showed the ability to tackle that problem [30,31]. In this study, everyday reminders to exercise via push notifications increased adherence to exercise compared to the control group. In the control group, we showed a link between higher initial disability and higher adherence to HEP. This link was not found in the intervention group. So, participants with a higher initial disability and who are at risk for developing chronic pain and disability [32,33] were adherent in both groups. The intervention increased adherence in participants with a lower initial disability and who already had a good recovery trajectory [6]. This could explain why increased adherence in the intervention group did not lead to an additional decrease in disability.

Psychological factors are often described to negatively impact recovery after a whiplash injury [32,33]. However, our study did not show this connection. Moreover, our results showed that pain catastrophizing correlated with actual disability levels. In both groups, catastrophizing decreased six months after the injury when disability levels decreased. However, the change in pain catastrophizing was significant only in the WIApp group. This is in accordance with Campbell et al. 2018 [34], who did not find evidence of pain catastrophizing negatively impacting recovery after a whiplash injury.

A work limitation is largely prevalent after a whiplash injury [35], and many people with injuries have a lower ability to work six months after their injury [36]. In our study, 8 out of 10 participants in the intervention and 9 out of 10 in the control group reported initial absenteeism, with a lower rate of work-disabled participants in the intervention group. Also, the intervention group had a lower number of days of sick leave, but it was not statistically significant. This is in accordance with the paper by Brakenridge, et al. [37], which states that interventions following whiplash injury were not effective in decreasing days of sick leave. Although both groups showed significant improvements in presenteeism, almost one out of three were unable to perform their usual work six months after the injury.

Social functioning is also affected by the injury [38,39]. Both groups showed significant improvement in social functioning, and no difference was found between them. In both groups, three out of five participants reported rare or no limitations in social functioning six months after the injury.

Whiplash injury patients often sustain chronic pain and disability and do not fully recover [40]. Accordingly, three out of five participants reported partial recovery after six months, and no difference was found between groups.

It is known that whiplash injury negatively impacts a person’s HRQoL [41,42]. When analyzing HRQoL, both groups showed significant improvement in a six-month period. As stated before, no difference was found between the groups when the outcomes were analyzed individually (physical health, mental health, work limitation, and social functioning). However, when united in HRQoL, the mobile app group showed a superior improvement. Similarly, in a study by Chen et al. [43], telerehabilitation increased HRQoL but did not affect physical functioning in patients with knee osteoarthritis.

## 5. Strengths and Limitations

The main strength of the study is the methodology—an RCT comparing the effectiveness of mobile app intervention in increasing adherence to HEP against written and illustrated material given to the whiplash injury participant. The methodology was guided by CONSORT recommendations, and all outcomes were reported according to CATWAD. Generally, a low dropout rate was observed (9/68, 13.2%), with only one participant dropping out after the allocation. Due to a low drop-out rate and increased adherence to HEP in the WIApp group, we assume the app is suitable for every smartphone user who has sustained a whiplash injury. There are a few limitations that need reflecting upon. First, blinding of the patients and outcome investigators could not be achieved due to the nature of the intervention. Second, all outcome measures were patient-reported. Although they are the current golden standard for evaluation and follow-up of a whiplash injury, they can be subject to malingering [44]. Third, the follow-up period was six months. Therefore, further studies are needed to evaluate the long-term outcomes between the groups.

## 6. Conclusions

By adding mobile app intervention, we increased adherence to HEP, but that did not have an added impact on disability and pain. However, in the intervention group, lowered pain catastrophizing and increased HRQoL were observed. In this study, we focused on adherence, so there was no difference in exercise presentation between groups. In the future, content could be added to the app to be more interactive and to provide educational materials, videos of the exercises, and progression feedback. To maximize the apps’ potential, further studies could investigate how more individualized exercise programs impact recovery in high-risk participants. These participants could be reassessed in real time, and treatment plans could be retailored as needed.

## Figures and Tables

**Table 1:** Baseline characteristics of participants.

	Total (n = 68)	WIApp (n = 29)	Control (n = 30)
Age, median [IQR]	38 [25–50]	37 [24–51]	35 [25–52]
Female, n [%]	43 [63]	19 [66]	17 [57]
Marital status, n [%]			
Single	34 [50]	12 [41]	15 [50]
Married	28 [41]	14 [48]	14 [47]
Divorced	5 [7]	3 [10]	0 [0]
Widowed	1 [2]	0 [0]	1 [3]
Education level, n [%]			
Elementary education	1 [1]	1 [3]	0 [0]
Secondary education	39 [57]	18 [62]	17 [57]
Bachelor’s level	11 [16]	5 [17]	5 [17]
Master’s/ Doctoral	17 [25]	5 [17]	8 [27]
Employed, n [%]	49 [72]	19 [66]	22 [73]
Initial absence from work, n [%]	42 [86] *	15 [79] *	20 [91] *
Time from accident, median [IQR]	29 [22–46]	36 [23–48.5]	27 [22–42.5]
Doctor’s office visits, mean ± SD	3.6 ± 0.96	3.8 ± 1.13	3.5 ± 0.81
analgesics use, n [%]	52 [76]	23 [79]	25 [83]

* Percentage of employed participants. WIApp—Whiplash mobile application, Intervention group.

**Table 2:** Primary and secondary outcomes at baseline and six-month follow-up for both groups.

	WIApp (n = 29)			Control (n = 30)			WIApp (n = 29)	Control (n = 30)	
	Initial	Final	*p*	Initial	Final	*p*	∆ = |Final − Initial|		*p*
Adherence, median [IQR]		3 [2–4]			2 [2–3]				0.005
NDI%, median [IQR]	38 [26–43]	16 [4–27]	<0.001	35 [26–47]	17 [9–27]	<0.001	20 [10–27]	17 [6–25]	0.516
Perceived recovery (full, partial, not), n [%]		11 [38], 16 [55], 2 [7]			12 [40], 16 [57], 1 [3]				0.823
PCS (significant/insignificant), n [%]	12 [41] vs 17 [59]	3 [10] vs. 26 [90]	0.010	* 9 [31] vs. 20 [69]	4 [13] vs. 26 [87]	0.120			
SF12, median [IQR]	40 [36–50]	60 [48–83]	<0.001	49 [36–57]	66 [45–78]	0.001	20 [6–36]	15 [9–23]	0.038
VAS pain, mean ± SD	5.4 ± 1.6	2.4 ± 2.6	<0.001	5.8 ± 1.7	2.9 ± 2.3	<0.001	1.45 ± 2.4	1.45 ± 1.95	0.900
SFS, median [IQR]	3 [3–4]	4 [3–4.5]	0.003	3 [2.75–4]	4 [3–4]	0.030	1 [0–1]	1 [0–1]	0.970
WLS, median [IQR]	3 [2–3.5]	2 [1–2.5]	0.003	3 [2–4]	2 [1–3]	0.004	1 [0–1.5]	1 [0–2]	0.770
Work time loss, median [IQR]		12 [0–58]			24 [10–70]				0.100

* Missing data for one participant who did not report PCS (valid percent reported). WIApp—Whiplash mobile application, Intervention group; NDI—Neck Disability Index; PCS—Pain Catastrophizing Scale; SF-12—Short Form Survey 12; SFS—Social Functioning Scale (1—constant limitation to 5—no limitation); WLS—Work Limitation Scale (1—no limitation to 6—complete limitation).

**Table 3:** Participants divided into outcome categories.

	Initially		After 6 Months	
	WIApp (n = 29)	Control (n = 30)	WIApp (n = 29)	Control (n = 30)
Adherence to exercise, n [%]				
Never			2 [7]	1 [3]
Occasionally			6 [21]	17 [57]
2–4 sessions/week			10 [34]	11 [37]
≥5 sessions/week			11 [38]	1 [5]
Neck Disability Index%, n [%]				
no disability (0–8%)			10 [34]	8 [27]
mild disability (10–28%)	8 [28]	10 [33]	14 [48]	16 [53]
moderate disability (30–48%)	15 [52]	13 [43]	4 [14]	3 [10]
severe disability (50–68%)	5 [17]	7 [23]	1 [3]	2 [7]
complete disability (>70%)	1 [3]			1 [3]
VAS pain (0–10), n [%]				
no pain (0)			9 [31]	5 [17]
mild pain (1–3)	2 [7]	4 [13]	13 [45]	15 [50]
moderate pain (4–6)	22 [76]	14 [47]	4 [14]	8 [27]
severe pain (7–10)	5 [17]	12 [40]	3 [10]	2 [7]
Social Functioning Scale (limitation), n [%]				
all of the time	1 [3]			
most of the time	5 [17]	7 [23]		1 [3]
some of the time	13 [45]	14 [47]	10 [34]	11 [37]
a little of the time	8 [28]	9 [30]	12 [41]	13 [43]
none of the time	2 [7]		7 [24]	5 [17]
Work Limitation Scale, n [%]				
No work limitation	1 [3]	1 [3]	11 [38]	9 [30]
I can do only my usual work	9 [31]	8 [27]	11 [38]	12 [40]
I can do most of my usual work, but no more	12 [41]	13 [43]	6 [21]	8 [27]
I can’t do my usual work	5 [17]	7 [23]	1 [3]	1 [3]
I can hardly do any work at all	1 [3]	0 [0]		
I can’t do any work at all	1 [3]	1 [3]		

WIApp—Whiplash mobile application, Intervention group.

**Table 4:** Estimates of model variables’ effects on adherence.

Variable	Effect Size (β)	Standard Error	*p* Value
Intercept	2.593	0.4156	<0.0001
NDI initially (%)	−0.006714	0.008337	0.4242
Pain (VAS) initially	−0.05571	0.06781	0.4150
PCS initially	0.01584	0.008822	0.0782
Intervention (app.)	0.5883	0.2082	0.0066

NDI—Neck Disability Index; PCS—Pain Catastrophizing Scale.

## Data Availability

Raw data can be provided upon request.

## Supplementary Materials

The following supporting information can be downloaded at https://www.mdpi.com/article/10.3390/diagnostics14232729/s1, Figure S1. Participant timeline and data collection method.
