# Supplementary material for: Mobile App Intervention Increases Adherence to Home Exercise Program After Whiplash Injury—A Randomized Controlled Trial (RCT)
Source: Diagnostics (Basel). 2024 Dec 4;14(23):2729. doi: 10.3390/diagnostics14232729 (PMC11640009; doi:10.3390/diagnostics14232729)
Supplement: Supplementary file 1 [file diagnostics-14-02729-s001.zip › diagnostics-3339312-supplementary.pdf]

Supplementary Material

	STUDY PERIOD		
	Enrolment	Allocation	Close-out
**TIMEPOINT**	$-t_1$	0	t_1 6 months
**Enrolment:**			
Eligibility screen	X		
Informed consent	X		
Allocation		X	
**INTERVENTION**			
Intervention group (standard care + mobile app)			
Control group (Standard care)			
**ASSESSMENTS**			
**Demographic data:**			
Age, gender, marital status, education level	X		
Employment status	X		X
Work-time loss	X		X
Other medical and treatment-related data	X		
**Self-reported data:**			
EAS			X
NDI	X		X
PRS	X		X
PCS	X		X
WLS	X		X
VAS of pain	X		X
SF-12	x		X

Standard care:
Mobile app:

Supplementary Figure S1. Participant timeline and data collection method.
